# Detecting Elevated Air Pollution Levels by Monitoring Web Search Queries: Algorithm Development and Validation

**DOI:** 10.2196/23422

**Published:** 2022-12-19

**Authors:** Chen Lin, Safoora Yousefi, Elvis Kahoro, Payam Karisani, Donghai Liang, Jeremy Sarnat, Eugene Agichtein

**Affiliations:** 1 Department of Computer Science Emory University Atlanta, GA United States; 2 Department of Computer Science Pomona College Claremont, CA United States; 3 Department of Environmental Health Emory University Atlanta, GA United States

**Keywords:** nowcasting of air pollution, web-based public health surveillance, neural network sequence modeling, search engine log analysis, air pollution exposure assessment, mobile phone

## Abstract

**Background:**

Real-time air pollution monitoring is a valuable tool for public health and environmental surveillance. In recent years, there has been a dramatic increase in air pollution forecasting and monitoring research using artificial neural networks. Most prior work relied on modeling pollutant concentrations collected from ground-based monitors and meteorological data for long-term forecasting of outdoor ozone (O_3_), oxides of nitrogen, and fine particulate matter (PM_2.5_). Given that traditional, highly sophisticated air quality monitors are expensive and not universally available, these models cannot adequately serve those not living near pollutant monitoring sites. Furthermore, because prior models were built based on physical measurement data collected from sensors, they may not be suitable for predicting the public health effects of pollution exposure.

**Objective:**

This study aimed to develop and validate models to *nowcast* the observed pollution levels using web search data, which are publicly available in near real time from major search engines.

**Methods:**

We developed novel machine learning–based models using both traditional supervised classification methods and state-of-the-art deep learning methods to detect elevated air pollution levels at the US city level by using generally available meteorological data and aggregate web-based search volume data derived from Google Trends. We validated the performance of these methods by predicting 3 critical air pollutants (O_3_, nitrogen dioxide, and PM_2.5_) across 10 major US metropolitan statistical areas in 2017 and 2018. We also explore different variations of the long short-term memory model and propose a novel search term dictionary learner-long short-term memory model to learn sequential patterns across multiple search terms for prediction.

**Results:**

The top-performing model was a deep neural sequence model long short-term memory, using meteorological and web search data, and reached an accuracy of 0.82 (*F*_1_-score 0.51) for O_3,_ 0.74 (*F*_1_-score 0.41) for nitrogen dioxide, and 0.85 (*F*_1_-score 0.27) for PM_2.5_, when used for detecting elevated pollution levels. Compared with using only meteorological data, the proposed method achieved superior accuracy by incorporating web search data.

**Conclusions:**

The results show that incorporating web search data with meteorological data improves the nowcasting performance for all 3 pollutants and suggest promising novel applications for tracking global physical phenomena using web search data.

## Introduction

### Background

Web-based crowd surveillance has been used to track emergent risks to public health [1-3]. Most commonly, these efforts involve the collection of web-based search queries to document acute changes in the incidence or symptom occurrence of primary infectious disease agents, such as influenza [4-7], Ebola [8], dengue fever [9], and COVID-19 [10]. These methods have the potential to provide public health and medical professionals with benefits over traditional health surveillance and environmental epidemiology in their ability to capture both personal exposures and response dynamics at more sensitive spatial and temporal scales [2].

Despite the promise of these approaches for infectious diseases, only a limited number of studies have examined how crowd surveillance approaches can be used to track environmental exposures and, less frequently, responses to noninfectious environment-mediated disease processes [11-13]. The global burden of disease attributable to outdoor and indoor air pollution has been quantified by recent efforts and has increased public awareness of the severity of this public health crisis worldwide [14]. Therefore, urban air pollution provides a key test case for the evaluation of web-based surveillance approaches for noninfectious environmental risks. The web-based surveillance approach is distinct from traditional approaches for measuring urban air pollution exposure. Therefore, it could possibly serve as a substitute to or complement the existing approaches. Traditional indicators of air pollution exposure, namely, concentrations measured at ambient monitoring sites, are widely used to assess the health effects associated with air pollution in epidemiological studies. However, the use of ambient monitoring measurements as surrogates of exposure may result in the misclassification of health responses and potential risks, especially for those not living near pollutant monitoring sites [15-17]. Moreover, ambient monitoring, by design, provides information on measured outdoor pollutant concentrations and may not necessarily reflect accurate personal exposures for individuals spending most of their time indoors or for those with preexisting biological susceptibility to air pollution. Several recent studies have focused on using smartphones within distributed air pollution sensing networks, where users record and upload local air pollution conditions to crowd-generated, geospatially refined pollution maps [11-13]. These studies demonstrate the feasibility of web-based crowd-generated participation in projects predicted on urban air pollution awareness.

To the best of our knowledge, few studies have investigated the feasibility of using web search data to produce accurate “nowcasts” of urban air pollution levels in real time. Conducting accurate predictions using web search data is a challenging task with 2 major challenges. The first is the selection of search terms to comprehensively capture people’s responses. Several approaches have been proposed to select search terms. For example, some studies preliminarily prepare keywords related to the target disease and then use these keywords to filter the search terms, which is often difficult because finding related keywords could be difficult for some diseases or be costly when conducting for multiple diseases. The second is the selection of the appropriate models. Although the literature on data-driven nowcasting methods for estimating infectious disease activity is well developed from an epidemiological standpoint, the machine learning methods used lag behind the state-of-the-art methods. The nowcasting models introduced to date mainly use variations of regularized linear regressions or, less often, random forests (RFs) or support vector machines. From a machine learning perspective, the problem of disease activity estimation is most suited to a more sophisticated and time series–specific model architecture. Because of the growing volume of recorded environment-mediated disease data, the use of recurrent neural networks (RNNs) and, more specifically, their variants long short-term memory (LSTM) and gated recurrent unit networks is increasingly feasible. The vanilla LSTM model makes predictions solely relying on the time series of the search activity while ignoring the semantic information in the search query phrases. Previous studies have pointed out that search queries could be semantically related, and ignoring their correlation would lead to a decrease in model performance [18,19]. Recent advances in natural language processing have led to the development of a technique called word embeddings to represent the semantic information in phrases, and fine-tuning of word embeddings has been encouraged for downstream tasks (Wu, Y, unpublished data, September 2016) [20-22]. However, there is still a lack of knowledge on incorporating both the semantic information of search queries and time series of search activities to make predictions.

### Objectives

In this study, we investigate web search data as an important source of a web-based crowd-based indicator. As web search data are free and broadly accessible, we posit that they could serve as a scalable means of tracking urban air pollution exposures and corresponding population-level health responses. To measure search interest, we used the freely accessible Google Trends service, which reports aggregate search volume data at a city-level geographical resolution. For this analysis, we use known health end point terms and topics, such as “difficulty breathing,” and observations (eg, “haze”) suggested by public health researchers, augmented by automatic term expansion based on semantic and temporal correlations, to estimate the levels of search activities related to air pollution, and ultimately to predict whether the pollution levels were elevated [23,24].

Compared with existing air pollution classification models, this study explores the use of web search anomalies as an auxiliary signal to detect air pollution. We compared our approach with the state-of-the-art physical sensor–based models that incorporate various pollutant covariates such as historical pollutant concentrations and meteorological data [25]. Using web search data for prediction introduces several challenges, including an unclear relationship between search interest and pollution levels and the trade-off between model complexity and convergence for the inclusion of web search data in a data-deficient scenario.

In summary, our contributions are as follows:

- We proposed a novel search term dictionary learner-LSTM (DL-LSTM) model to learn sequential patterns from broad historical records of web search data for air pollution nowcasting.
- We compared the DL-LSTM models with a variety of baseline models on the efficacy of using web search data to indicate exposure to a noninfectious environmental stressor (ie, air pollution) and demonstrate that the proposed models are effective across different experimental settings.
- We evaluated the efficacy of combining web search data and meteorological data for air pollution prediction and showed that the inclusion of web search data improves the prediction accuracy and provides a promising substitute when historical pollutant data are unavailable.

## Methods

We now describe the methodology. First, we formalize our problem setting, then describe the data, and then introduce our modeling approaches.

### Problem Statement

We formalized this task as a classification problem and adapted state-of-the-art machine learning models. We constructed a multivariate autoregressive model and an RF model fit on historical air pollutant concentrations as well as search and meteorological data as baseline models. We evaluated the performance of our proposed models (described below) in comparison with the baselines in terms of prediction accuracy and other standard classification prediction metrics.

### Ethical Considerations

The data available to the public are not individually identifiable and therefore analysis does not involve human subjects. The International Review Board (IRB) recognizes that the analysis of de-identified, publicly available data does not constitute human subjects research and therefore does not require IRB review.

### Data Collection

We collected daily air pollutant concentration data as well as temperature and relative humidity in the 10 largest US. metropolitan statistical areas (MSAs) from January 2007 to December 2018. We focused on 3 air pollutants: ozone (O_3_), nitrogen dioxide (NO_2_), and fine particulate matter (PM_2.5_). The in-situ pollutant concentrations and meteorological data such as temperature, relative humidity, and dew point temperature were retrieved from the US Environmental Protection Agency, Air Quality System, and AirNow database. To create a single daily pollutant concentration for each city, we used the median pollutant concentration from all available monitoring sites within each city to avoid outlier bias.

We collected the daily search frequency of pollution-related terms from Google Trends for the same 12-year period and cities. We created a curated list of 152 pollution-related terms based on our previous air pollution epidemiology studies and in reviewing the environmental health literature [14,26-30], and we downloaded the reports of trending results terms using PyTrends [31]. For each PyTrends request, we downloaded the search history of pollution-related terms over a 6-month window with 1 overlapping month for calibration. PyTrends provided us with a search frequency scaled on a range of 0 to 100 based on a topic’s proportion to all searches on all topics. Because of the PyTrends restriction, we downloaded the reports of trending results multiple times, and the search frequencies were scaled separately in each 6-month window, which required us to calibrate the search frequency for the 12-year period. We calibrated the search frequencies by joining the search logs on the overlapping periods (1 out of 6 months) for intercalibration [32].

We investigated the available input features from meteorological data (temperature and relative humidity), historical pollutant concentrations, and web search data (Table 1).

**Table 1:** Input features calculated per time step in the input sequence.

Input feature	Feature transformation
Meteorological data (Met^a^)	Maximum temperature (Temp_max^b^) Mean temperature (Temp_mean^c^) Relative humidity (humidity) Square of Temp_mean Cube of Temp_mean Square of humidity Cube of humidity Dew point temperature
Pollutant concentration (Pol^d^)	Concentration on day t-7^e^ Concentration on day t-6^e^ Concentration on day t-5^e^ Concentration on day t-4^e^ Concentration on day t-3^e^ Concentration on day t-2^e^ Concentration on day t-1^e^
Search	Search volumes of search terms

^a^Met_:_ meteorological data.

^b^Temp_max: maximum temperature

^c^Temp_mean: mean temperature

^d^Pol_:_ pollutant concentration.

^e^Day t-7,..., t-1: days preceding the prediction day t.

### Missing Data Imputation and Normalization

Smoothing and interpolation are simple and efficient data imputation methods [33], and we applied linear interpolation to fill the missing data in historical pollutant concentration, temperature, and humidity, with a rolling window size of 3. To fill in the missing data in infrequent search terms for which Google Trends does not return a count, we used random numbers close to 0 (e^-10^~e^-5^). We normalized all the input features to standard scores by subtracting their mean values and dividing them by the respective SDs.

### Search Term Expansion

As web-based search queries may reflect individual exposure to ambient air pollution, the seed terms were mostly related to symptoms, observations, and emission sources (Table S1 in Multimedia Appendix 1). However, because an exhaustive list of user queries was not available, reliance on only expert-generated seed words may result in poor prediction because of the high mismatch rate between the user queries and our expected search words.

Query expansion is a common approach for resolving this discrepancy. A recent study [18] showed that the initial set of seed words could be effectively expanded through semantic and temporal correlations. Thus, for each seed word, we used Google Correlate [34] to retrieve the top 100 correlated query terms. Then, we used the pretrained word2vec model [21] to retrieve the vector representation of each query; phrases were mapped to the centroid of the constituent terms. A utility score was calculated for each candidate query by measuring the maximum cosine similarity between the query and seed words. Queries with a high utility score were retained, and the remaining queries were eliminated, and we empirically set the utility cutoff to 0.55. This method expanded the set of search terms for the 152 search terms to track (Table S2 in Multimedia Appendix 1).

### Modeling and Evaluation

#### Problem Definition

Given sequences of physical sensor data P = [p_t-L,_..., p_t-1_]^T^ with the dimension of L times d_p_, and search interest data S = [s_t-L+2_,..., s_t+1_]^T^ with the dimension of L times d_s_, the task is to classify day *t* as *polluted* or not, where a positive class label indicates that the air pollution was above a predefined threshold. L denotes the sequence length, and d_p_ and d_s_ are the number of physical sensor features and the number of search-related terms, respectively.

#### Autoregressive and RF Classification Models

Previous work has shown that simple autoregressive models using web search data can generate nowcast estimates for influenza-like illnesses at the US national level [19]. We adapted autoregressive models with a logistic regression (LR) classifier for classification purposes. Furthermore, we applied elastic net regularization, which is a linear combination of *l_1_* and *l_2_* regularization, as proposed in previous studies [18,19]. LR+Elastic Net was implemented using the Python *scikit-learn* package, using cross-validation to set the model’s hyperparameters to maximize the *F*_1_-score on the validation set, with class_weight set to “balanced.”

RF is an ensemble learning model that is robust against overfitting and provides a strong baseline for the development of nonlinear predictive models [35]. We used the *scikit-learn* implementation of RFs. The number of trees and maximum depth of individual trees were selected to maximize the *F*_1_-score on the validation set, with balanced class_weight for positive and negative samples.

#### LSTM and Its Variants

**Figure 1:**
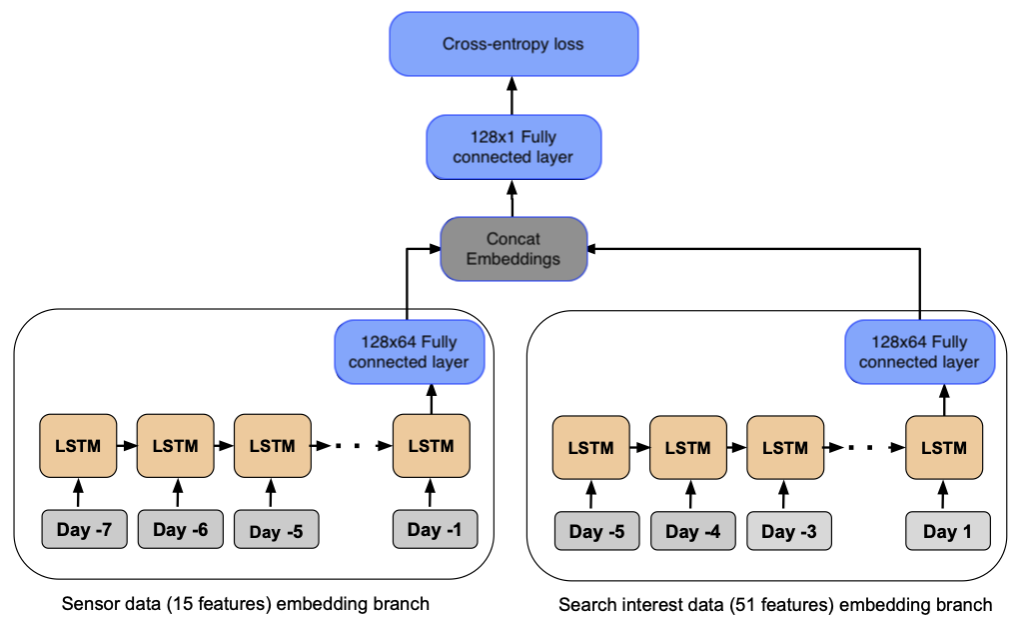
The architecture of the long short-term memory (LSTM) model.

LSTM units [36] are RNN models designed for sequence modeling, which can learn nonlinear relationships in time series data [37]. First, we describe a baseline LSTM model with 2 subnetworks to separate the search data and meteorological data. As shown in Figure 1, there are 4 layers in the model, that is, the sequence embedding layer, LSTM layer, fully connected hidden layer, and output layer [38].

In the left subnetwork of the LSTM model with search data as input, we propose 2 methods for capturing semantic information in search terms. The first is the LSTM semantic model (GloVe [Global Vectors for Word Representation]; LSTM-GloVe). As a variant of the vanilla LSTM model, for the sequence embedding layer of the right subnetwork in Figure 1, we introduce the matrix multiplication operation to project the search values of search terms to their semantic embedding space (GloVe embeddings), as shown in equation 1.

Given the search interest data S = [s_1_,..., s_7_]^T^ with the dimension of 7 times d_s_, and their GloVe embedding G = [g_1_,..., g_dg_] with the dimension of d_s_ times d_g_, where d_g_ = 50 (GloVe 50-dimensional word vectors trained on tweets [22]). The matrix multiplication operation is defined as

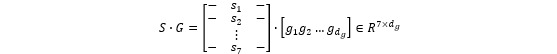



Specifically, the tensor generated by the matrix multiplication operation was then fed into the LSTM layer for further calculations. This matrix multiplication is designed specifically for the model consistency problem when introducing collinear predictors after search term expansion (STE).

**Figure 2:**
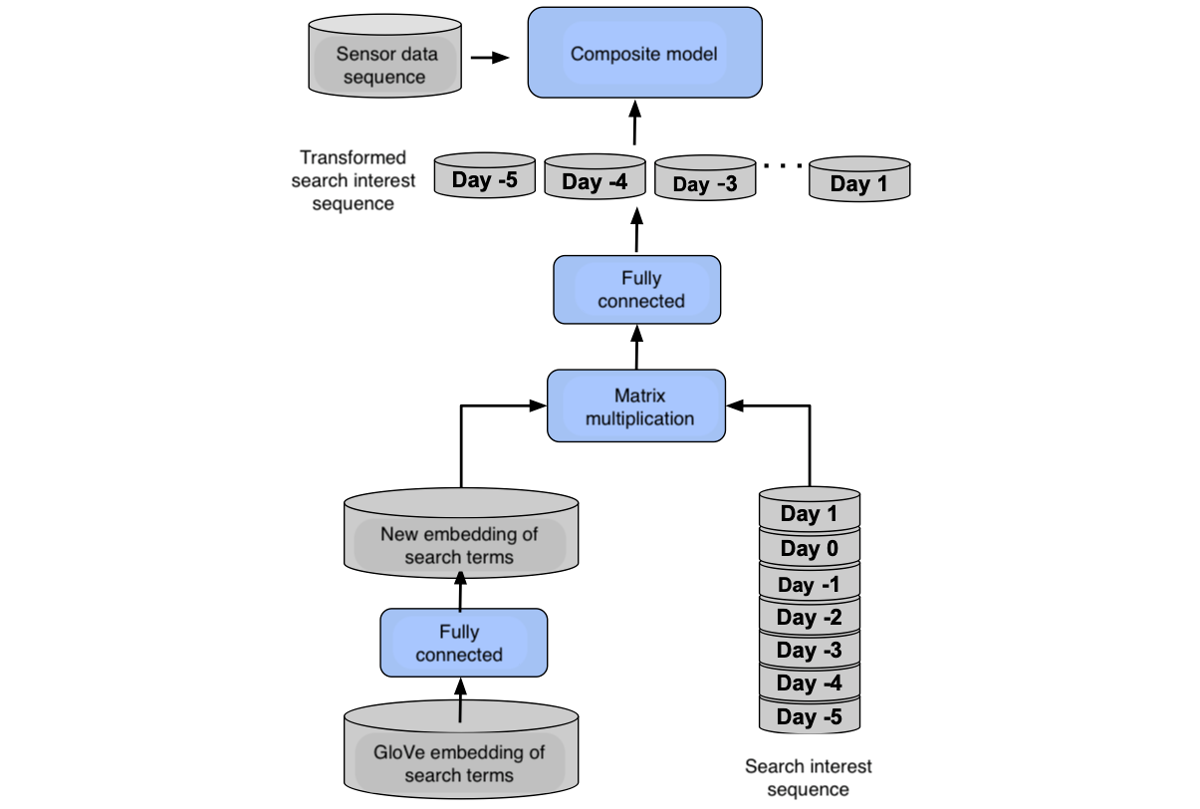
The architecture of the dictionary learner-long short-term memory model.

The second variation of the LSTM model is the DL-LSTM model, which is theoretically based on the idea of matrix multiplication, as shown in LSTM-GloVe. However, instead of directly applying the GloVe embedding for matrix multiplication, it introduces the fine-tuning of the word embeddings via a *d_g_* by *d_e_* rectified linear unit–activated fully connected layer. As shown in Figure 2, the rectified linear unit–activated fully connected layer was applied to the initial GloVe embedding, where *d_e_*=100 is the size of the new embedding. In this architecture, the GloVe 50-dimensional word vectors are used to initialize the search term embedding dictionary, and the matrix multiplication operation is used to transform the input embedding of search terms into the semantic embedding space [39].

In summary, we evaluate the following models in this paper:

- LR: LR is LR classifier with elastic net regularization.
- RF: RF is RF classifier with the number of trees and maximum depth tuned for prediction.
- LSTM: The baseline LSTM model, as shown in Figure 1, combines physical sensor features, if available, with the search interest volume data directly, providing a direct adaptation of RNNs to this problem without any problem-specific extensions.
- LSTM-GloVe: LSTM semantic model is a variant of the LSTM model as described in equation 1, where we control the input of search interest data (ie, 51 seed search terms vs 152 terms after STE) in this model. We refer to the variants as *LSTM-GloVe* and *LSTM-GloVe with [w/] STE*, respectively.
- DL-LSTM: The DL-LSTM model is shown in Figure 2. We control the input of the search interest data (ie, 51 seed search terms vs 152 terms after STE) in this model and refer to the variants as *DL-LSTM* and *DL-LSTM w/STE*, respectively.

### Validation

To tune the model parameters and validate the model performance, we split the available data into training (from January 2007 to December 2014), validation (from January 2015 to December 2016), and testing (from January 2017 to December 2018) sets. This 8-year training period provides a broad history for learning the relationship between input and output variables, and the predictive models are evaluated based on their ability to make predictions for completely unseen periods. For evaluating our model, we made predictions for each day from January 2017 to December 2018 in the test data set. The distribution of the classes in the training, validation, and test data sets is presented in Table 2. Note that the positive and negative classes are heavily imbalanced, with positive classes comprising, for instance, only 16% of the training samples when PM_2.5_ is the target pollutant.

**Table 2:** The distribution of classes in the training, validation, and test sets.

Pollutant	Negative samples				Positive samples		
	Training	Validation	Test	Training		Validation	Test
O_3_^a^	24,322	6269	6311	4896		1038	982
NO_2_^b^	23,926	6119	6332	5292		1188	961
PM_2.5_^c^	24,297	6745	6757	4921		562	536

^a^O_3_: ozone.

^b^NO_2_: nitrogen dioxide.

^c^PM_2.5_: fine particulate matter.

### Evaluation Metrics

As we defined this task as a classification problem, we used the standard classification evaluation metrics. We report the accuracy and *F*_1_-score of the positive class (the harmonic mean of precision and recall) of the predictions as evaluation metrics for all models. Although accuracy measures the total fraction of correct predictions and could misrepresent model performance in the presence of heavily imbalanced classes, the *F*_1_-score considers class imbalance and is, therefore, a more appropriate metric for our problem.

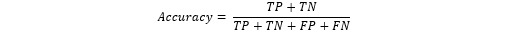

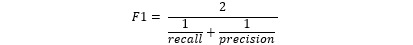





Where *TP*, *TN*, *FP*, and *FN* are the number of true positive samples, true negative samples, false positive samples, and false negative samples, respectively.

## Results

### Overview

In this section, we first present the findings of the data exploration. Next, we present the principal findings of this study.

### Insights From Collected Data

In this section, we describe the thresholds of abnormal air pollutant concentrations and present the lag between the search anomalies and air pollution.

#### Thresholds of Abnormal Air Pollutant Concentrations

**Figure 3:**
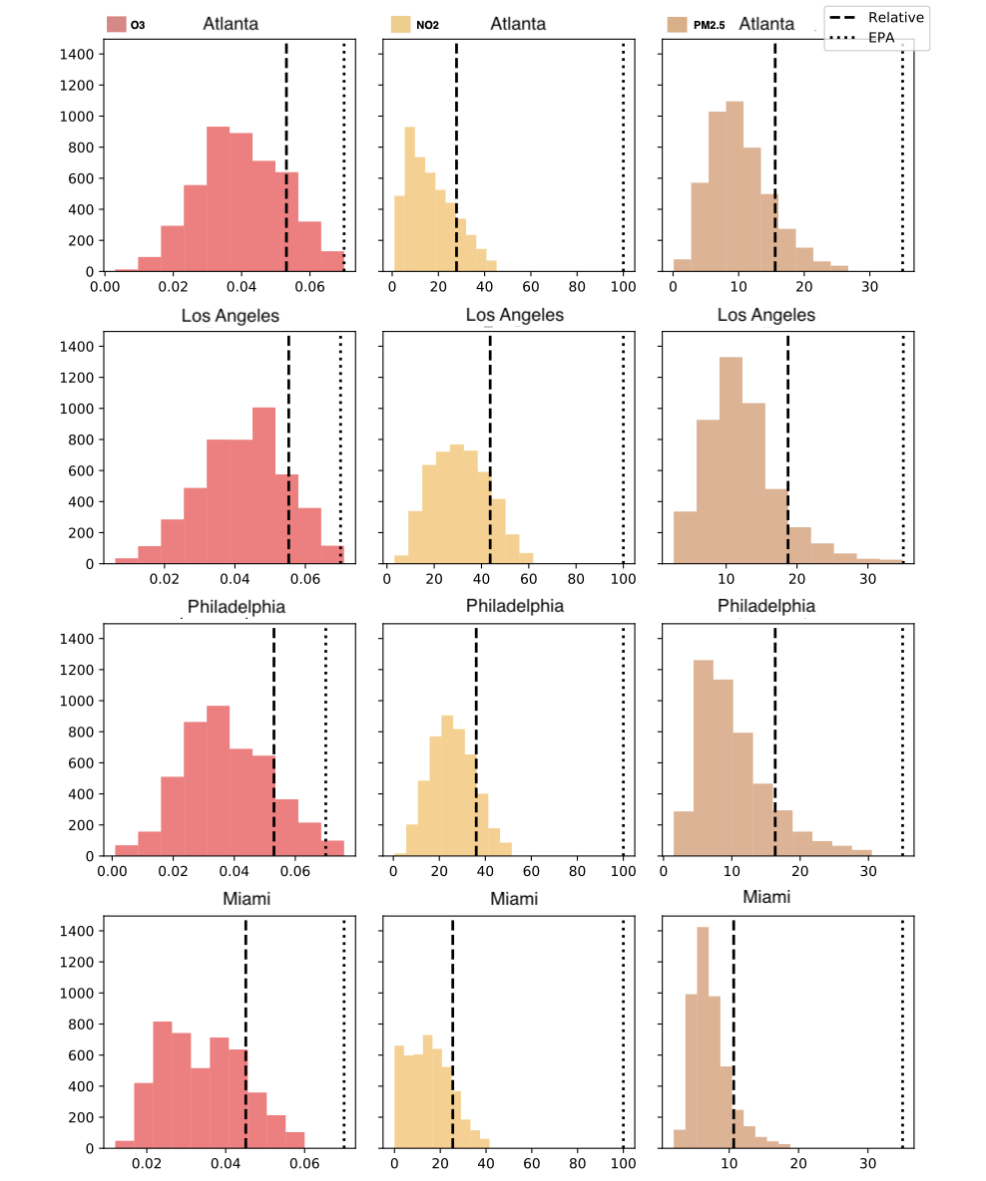
Distribution of pollution values for Atlanta, Los Angeles, Philadelphia, and Miami, with city-specific elevated pollution level (dashed line) and the general Environmental Protection Agency–mandated standard (dotted line), for ozone (O_3_; left column), nitrogen dioxide (NO_2_; middle column), and fine particulate matter (PM_2.5_; right column). EPA: Environmental Protection Agency.

The major MSAs chosen for this study have different distributions of pollutant concentrations over time and almost always fall below the Environmental Protection Agency standard 24-hour threshold (Figure 3). However, multiple studies have shown that even at low concentrations, chronic exposure to air pollution negatively affects human health [26,27]. Therefore, calibrating a meaningful threshold for each city, especially those with generally lower levels of air pollution (eg, Miami), may be critical for adequately protecting population health. A natural way to do this may be to set the threshold to 1 SD above the mean daily pollutant concentration within each city, which was adopted in this study. The input predictors were also normalized within each city to reflect the city-level dynamics. The resulting thresholds for the 3 pollutants and cities under investigation are reported in Table 3.

**Table 3:** Classification thresholds for 3 pollutants across 10 major metropolitan statistical areas in the United States.

Pollutant	Los Angeles	District of Columbia	Philadelphia	Dallas	Atlanta	Boston	New York	Miami	Chicago	Houston
O_3_^a^ (ppb^b^)	55	54	53	53	53	48	49	45	49	49
NO_2_^c^ (ppb)	43.7	38.1	36	25.2	27.8	30.7	45.3	25.5	43.7	27.7
PM_2.5_^d^ (µg/m^3^)	18.7	15.1	16.4	13.1	15.6	12.4	13.9	10.6	16.2	14.4

^a^O_3_: ozone.

^b^ppb: parts per billion.

^c^NO_2_: nitrogen dioxide.

^d^PM_2.5_: fine particulate matter.

#### Lag Between Search Anomalies and Air Pollution

**Figure 4:**
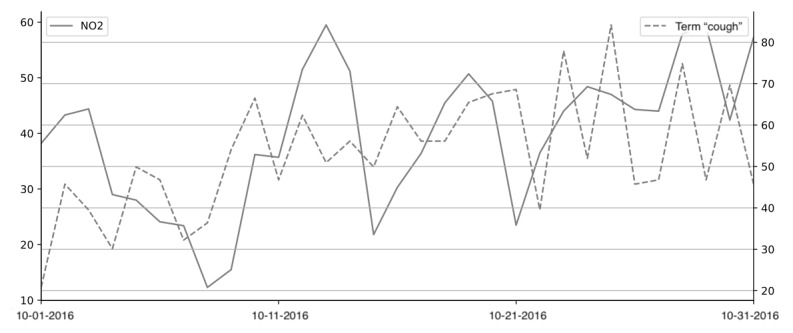
Daily nitrogen dioxide (NO_2_) levels and search interest for the term “cough” in October 2016 in Atlanta.

A previous study showed that there could be a lag between incident occurrence and Google search activity [40]. As shown in Figure 4, the normalized search frequency of the term “cough” is correlated with the concentration of NO_2_ in Atlanta with a certain lag of time. To determine the lag between elevated pollution levels and consequent pollution-related searches, the mean absolute Spearman correlation between pollutant concentrations and search interest data was calculated and shifted forward in time for 0, 1, 2, and 3 days. As shown in Table 4, for O_3_ and PM_2.5_, the mean absolute Spearman correlation increased with an increase in the shifted days. Considering that the task aimed to detect elevated pollution levels as soon as possible, a lag of 1 day was applied to search data. In other words, the search interest data from the current day were used to estimate whether air pollution was elevated on the previous day.

**Table 4:** Cross-correlation of top 5 search terms with different lags for 3 pollutants in the Atlanta metropolitan area in 2016 (N=366).

Pollutant	Lag=0; search term (Spearman correlation)	*P* value	Lag=1; search term (Spearman correlation)	*P* value	Lag=2; search term (Spearman correlation)	*P* value	Lag=3; search term (Spearman correlation)	*P* value
**O_3_^a^**								
	Cough (−0.34)	<.001	Cough (−0.38)	<.001	Cough (−0.41)	<.001	Cough (−0.41)	<.001
	Bronchitis (−0.31)	<.001	Bronchitis (−0.32)	<.001	Bronchitis (−0.33)	<.001	Bronchitis (−0.35)	<.001
	Traffic (0.26)	<.001	Traffic (0.27)	<.001	Traffic (0.26)	<.001	Smoke (0.24)	<.001
	Smoke (0.23)	<.001	Chest pain (−0.23)	<.001	Chest pain (−0.23)	<.001	Traffic (0.23)	<.001
	Snoring (0.22)	<.001	Snoring (0.22)	<.001	Smoke (0.22)	<.001	Chest pain (−0.22)	<.001
**NO_2_^b^**								
	Asthma (0.20)	<.001	Sulfate (0.20)	<.001	Sulfate (0.16)	.002	Cough (0.16)	.002
	Sulfate (0.19)	<.001	Bronchitis (0.16)	.002	Bronchitis (0.15)	.005	COPD^c^ (−0.16)	.003
	Cough (0.17)	<.001	Inhaler (0.15)	.005	Cough (0.14)	.008	Bronchitis (0.14)	.008
	Bronchitis (0.17)	.001	Cough (0.14)	.006	Inhaler (0.11)	.03	Wheezing (−0.12)	.02
	Inhaler (0.16)	.002	Difficulty breathing (−0.12)	.02	Headache (−0.11)	.03	Headache (−0.10)	.04
**PM_2.5_^d^**								
	Wildfires (0.14)	.009	COPD (−0.15)	.005	Air pollution (0.19)	<.001	Air pollution (0.18)	<.001
	COPD (−0.11)	.03	Wildfires (0.14)	.007	COPD (−0.17)	.001	COPD (−0.18)	<.001
	Snoring (0.11)	.03	Air pollution (0.14)	.008	Wildfires (0.14)	.009	Wildfires (0.15)	.004
	Inhaler (0.10)	.06	Asthma attack (0.11)	.04	Respiratory illness (0.10)	.05	Sulfate (−0.11)	.03
	Difficulty breathing (−0.09)	.08	Respiratory illness (0.10)	.05	Traffic (0.10)	.06	Traffic (0.11)	.04

^a^O_3_: ozone.

^b^NO_2_: nitrogen dioxide.

^c^COPD: chronic obstructive pulmonary disease.

^d^PM_2.5_: fine particulate matter.

### Evaluation Outcomes

In this section, we consider 3 conditions to evaluate the performance of using web search data to detect elevated pollution, that is, using only search data, using search data as auxiliary data for meteorological data, and using search data as auxiliary data for meteorological data and historical pollutant concentrations.

#### Using Only Search Data

For areas where ambient pollution monitoring is unavailable, investigating whether web search data can be used as the only signal for nowcasting elevated air pollution is a vital question. When relying only on search data for air pollution prediction, both the proposed DL-LSTM architecture and STE contribute to the improvement of prediction accuracy. As shown in the “Search” section of Table 5, the LSTM-based models exhibited superior accuracy over the baseline LR and RF models for O_3_ and NO_2._ For PM_2.5_, the proposed models did not perform better than the baseline LR or LSTM model because the validation and test data sets were heavily imbalanced (Table 5). The proposed DL-LSTM w/STE model achieved the highest *F*_1_-score (32.44% for O_3_ and 27.70% for NO_2_) for detecting O_3_ and NO_2_ pollution.

**Table 5:** Accuracy and *F*1-score of the logistic regression, random forest, and long short-term memory models for detecting elevated pollution across 10 major US cities, for varying input feature combinations: no prior knowledge, search data only (Search), meteorological data only (Met), meteorological data and search data (Met+Search), meteorological data and historical pollutant concentration (Met+Pol) and all input features (Met+Pol+Search).

Features and model		O_3_^a^, accuracy (*F*_1_-score; %)	NO_2_^b^, accuracy (*F*_1_-score; %)	PM_2.5_^c^, accuracy (*F*_1_-score; %)
**No prior knowledge**				
	All positives	13.46 (23.73)	13.18 (23.28)	7.35 (13.69)
	All negatives	86.54 (0.0)	86.82 (0.0)	92.65 (0.0)
	Random (prob of positive=0.5)	50.29 (20.63)	50.56 (20.68)	50.65 (12.67)
**Search**				
	LR^d^	36.93 (17.77)	53.97 (24.17)	78.29 (10.72)
	RF^e^	33.53 (23.36)	55.22 (18.1)	*92.65* ^f^ *(0.0)*
	LSTM^g^	46.73 (23.63)	69.68 (21.62)	89.96 (7.58)
	LSTM-GloVe^h^	53.23 (28.45)	63.44 (27.4)	90.09 (3.73)
	LSTM-GloVe w/STE^i^	69.17 (28.04)	46.85 (26.51)	91.73 (1.31)
	DL-LSTM^j^	62.46 (30.4)	65.99 (26.19)	88.61 (7.97)
	DL-LSTM w/STE	69.61 (32.44)	56.84 (27.7)	87.59 (6.99)
**Met**				
	LR	62.57 (39.81)	63.64 (37.25)	58.58 (22)
	RF	78.76 (50.59)	71.77 (39.88)	73.78 (24.67)
	LSTM	76.54 (48.29)	72.52 (41.27)	67.89 (24.69)
**Met+search**				
	LR	55.99 (36.56)	62 (36.25)	61.25 (21.5)
	RF	81.39 (45.35)	73.77 (38.71)	87.96 (23.78)
	LSTM	78.18 (47.65)	77.75 (40.31)	88.14 (21.29)
	LSTM-GloVe	80.04 (49.37)	72.75 (40.35)	85.38 (26.99)
	LSTM-GloVe w/STE	81.85 (50.71)	74.21 (41.49)	85.42 (26.13)
	DL-LSTM	77.97 (48.94)	74.81 (40.53)	84.94 (24.07)
	DL-LSTM w/STE	80.16 (49.32)	72.99 (40.34)	87.04 (21.32)
**Met+pol**				
	LR	67.38 (44.61)	70.05 (44.09)	74.45 (32.82)
	RF	82.81 (57.23)	80.35 (51.24)	86.45 (40.63)
	LSTM	86.97 (63.01)	84.64 (55.59)	85.25 (43.19)
**Met+pol+search**				
	LR	66.91 (43.71)	69.13 (43.6)	74.45 (32.82)
	RF	82.76 (55.91)	78.91 (47.72)	89.43 (37.57)
	LSTM	87.11 (61.54)	84.71 (54.02)	90.74 (44.81)
	LSTM-GloVe	87.94 (63.81)	82.98 (53.78)	88.19 (46.55)
	LSTM-GloVe w/STE	87.63 (63.83)	83.81 (54.59)	88.24 (46.51)
	DL-LSTM	87.30 (63.02)	82.65 (53.65)	89.66 (47.35)
	DL-LSTM w/STE	87.60 (63.61)	83.40 (53.58)	89.25 (46.59)

^a^O_3_: ozone.

^b^NO_2_: nitrogen dioxide.

^c^PM_2.5_: fine particulate matter.

^d^LR: logistic regression.

^e^RF: random forest.

^f^This high accuracy is simply due to class imbalance; this model always predicts negative class, and the corresponding *F*_1_-score is 0.

^g^LSTM: long short-term memory.

^h^GloVe: Global Vectors for Word Representation.

^i^STE: search term expansion.

^j^DL-LSTM: dictionary learner-long short-term memory.

#### Using Search Data and Meteorological Data

When meteorological data were available, we investigated the feasibility of using meteorological data with or without search activity data to nowcast air pollution under this condition. As shown in the “Met” and “Met+Search” sections of Table 5, the inclusion of web search data improves the nowcasting accuracy for all 3 pollutants. In addition, the LSTM-GloVe w/STE model achieved the highest *F*_1_-score (50.71% for O_3_ and 41.49% for NO_2_) for the detection of O_3_ and NO_2_ pollution. The LSTM-GloVe without STE model achieved the highest *F*_1_-score (26.99%) for detecting PM_2.5_ pollution.

#### Using Search Data, Meteorological Data, and Historical Pollutant Concentration

When historical pollution concentration is available, search activity data are added as auxiliary data to both meteorological data and historical pollution data. As shown in the “Met+Pol” and “Met+Pol+Search” sections of Table 5, the inclusion of web search data improves the nowcasting accuracy for O_3_ and PM_2.5_. However, for NO_2,_ the inclusion of web search data does not improve the nowcasting accuracy, which indicates that increases in NO_2_ concentrations may not be directly noticeable by people sufficiently to increase their search interest. This difference in the performance for different pollutants and locations merits further investigation.

### City-Level Analysis of O_3_ Pollution Prediction

We investigated the potential of using search interest and meteorological data to replace ground-based O_3_ sensor data for predicting O_3_ pollution in individual cities. As shown in Table 6, including search interest data (Met+Search) to augment purely meteorological data (Met) increases both the accuracy and *F*_1_-score metrics for most cities. Although these metrics do not reach performance when ground-level pollution sensors are available (Met+Pol), at least for two of the major MSAs (Philadelphia and Houston), search volume data indeed provides a useful alternative to pollution monitors, with only 1.6% and 0.14% degradation in accuracy, respectively. In addition, the differences in model performance across different cities indicate that web-based search patterns could vary from city to city. As shown in Table 7, the top 5 correlated terms differ across US cities over 10 years. The variation in search patterns could lead to degraded prediction performance in certain areas, leaving promising directions for improvement.

**Table 6:** City-level accuracy and *F*1-score for detecting elevated ozone pollution in 10 US cities, with Met (long short-term memory model), Met+Search (dictionary learner-long short-term memory w/search term expansion) and Met+Pol (long short-term memory model) as features.

Features			Los Angeles		District of Columbia		Philadelphia		Dallas		Atlanta		Boston		New York		Miami		Chicago		Houston
**Accuracy, %**																					
	Met^a^	72.6		77.4		83.29		83.42		83.56		75.62		68.36		58.09		76.71		85.89	
	Met+search	76.71		80.68		87.4		79.86		83.84		78.63		74.93		69.29		80		90.14	
	Met+pol^b^	85.89		86.99		89.04		89.04		88.22		84.66		86.85		82.02		86.85		90	
***F*_1_- score, %**																					
	Met	51.69		48.28		53.79		53.28		48.72		46.06		44.07		32.52		56.19		57.26	
	Met+search	54.3		50.53		58.56		41.9		42.72		48		47.86		35.84		57.56		59.09	
	Met+pol	68.11		60.58		64.29		64.6		56.12		55.56		63.64		55.48		70.73		67.26	

^a^Met: meteorological data.

^b^Pol: pollution data.

**Table 7:** Top 5 correlated search terms for ozone pollution in 10 US cities: January 1, 2010, to December 31, 2019.

City and search term			Spearman correlation (lag=1)
**Los Angeles**			
	Cough	−0.40	
	Bronchitis	−0.33	
	Wildfires	0.24	
	Traffic	0.14	
	Respiratory infection	−0.12	
**District of Columbia**			
	Bronchitis	−0.25	
	Cough	−0.25	
	Coughing	−0.19	
	Headache	−0.14	
	Wildfires	0.13	
**Philadelphia**			
	Cough	−0.33	
	Traffic	0.27	
	Bronchitis	−0.20	
	Organic carbon	−0.10	
	Respiratory infection	−0.09	
**Dallas**			
	Cough	−0.25	
	Bronchitis	−0.24	
	Ozone	0.17	
	Wildfires	0.15	
	Coughing	−0.14	
**Atlanta**			
	Bronchitis	−0.14	
	Cough	−0.11	
	Chest pain	−0.10	
	Respiratory infection	−0.09	
	Wheezing	−0.07	
**Boston**			
	Smoke	−0.11	
	Haze	−0.07	
	Code red	−0.06	
	Coughing	0.06	
	Smog	0.05	
**New York**			
	Bronchitis	−0.31	
	Traffic	0.29	
	Cough	−0.25	
	Wildfires	0.19	
	Wheezing	−0.15	
**Miami**			
	Bronchitis	0.14	
	Air pollution	0.13	
	Cough	0.13	
	Power plants	0.09	
	Nitrogen dioxide	0.08	
**Chicago**			
	Wildfires	0.18	
	Smoke	0.08	
	Shortness of breath	0.04	
	Heart murmur	0.04	
	Tail pipe	0.04	
**Houston**			
	Ozone	0.12	
	Air pollution	0.12	
	Asthma	0.06	
	Organic carbon	0.05	
	Wildfires	0.05	

### Sensitivity Analysis of Air Pollution Thresholds

**Figure 5:**
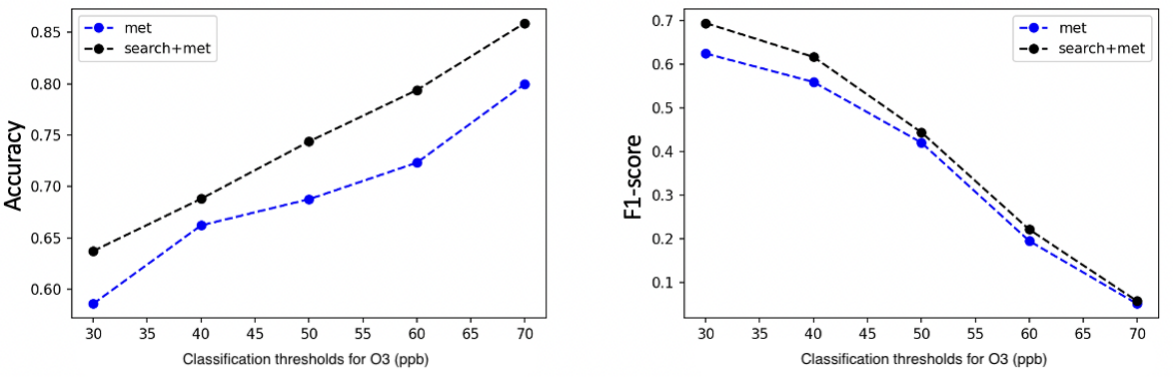
Accuracy (left figure) and *F*1-score (right figure) for detecting ozone (O_3_) pollution on various classification thresholds, with Met (long short-term memory model) and Met+Search (dictionary learner-long short-term memory w/search term expansion) as features. Met: meteorological data; ppb: parts per billion.

**Figure 6:**
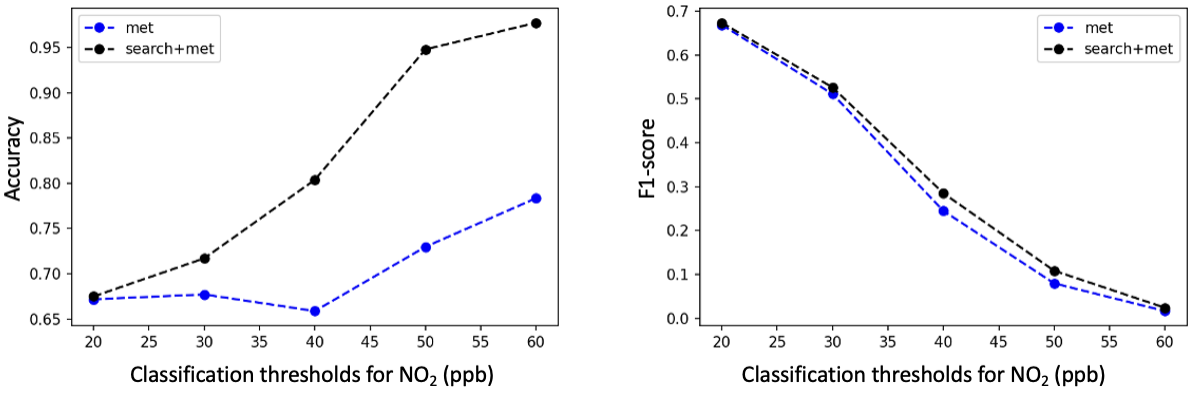
Accuracy (left figure) and *F*1-score (right figure) for detecting nitrogen dioxide (NO_2_) pollution on various classification thresholds, with Met (long short-term memory model) and Met+Search (dictionary learner-long short-term memory w/search term expansion) as features. Met: meteorological data; ppb: parts per billion.

**Figure 7:**
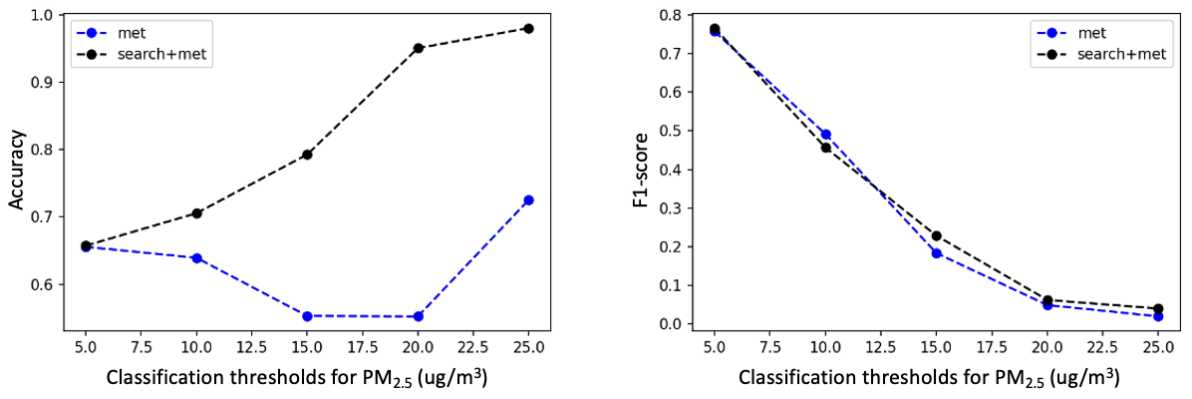
Accuracy (left figure) and *F*1-score (right figure) for detecting fine particulate matter (PM_2.5_) pollution on various classification thresholds, with Met (long short-term memory model) and Met+Search (dictionary learner-long short-term memory w/search term expansion) as features. Met: meteorological data.

Classification thresholds play an important role in our model. In this study, an SD threshold from the mean of the corresponding pollutants was used as a “probability threshold” to detect air pollution at a spatial-temporal resolution. However, the proposed method is sensitive to this threshold. We further investigated the performance of the proposed method using a variety of fixed classification thresholds. As shown in Figures 5-7, we fixed the classification thresholds for all 10 cities to detect O_3_, NO_2_, and PM_2.5_ pollutions. The results show that the meteorological and search data are complementary, and combining the search and meteorological data leads to better prediction performance for all classification thresholds.

## Discussion

### Principal Findings

In this study, we explored various existing air pollution prediction models and found that the use of a time series neural network approach achieved the highest predictive accuracy in most of our experiments. The results showed that the LSTM-based models achieved superior accuracy for the 3 air pollutants when both meteorological data and web search data were available. Furthermore, our results on the inclusion of web search data with meteorological data indicate that under short reporting delays, the LSTM models could provide highly accurate predictions compared with baseline models using meteorological and historical pollution concentration data.

**Figure 8:**
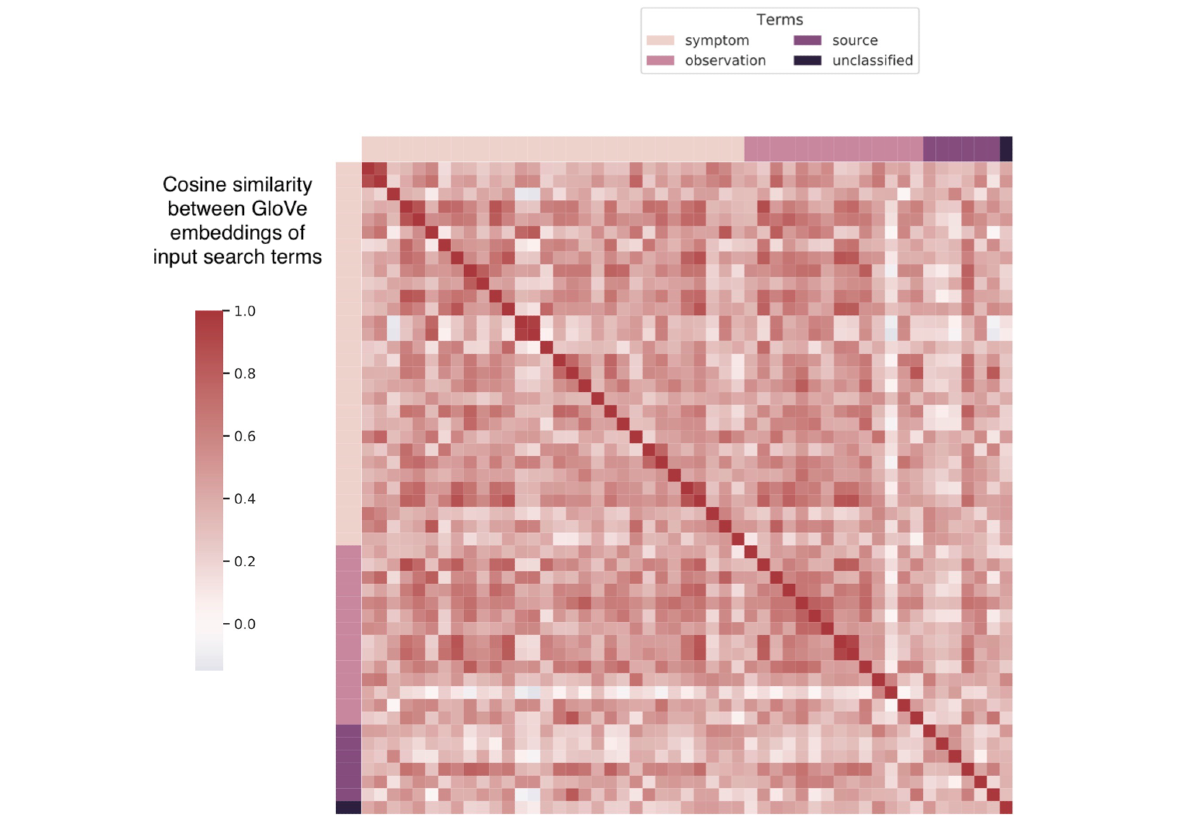
Cosine similarity between GloVe embeddings of seed search terms. GloVe: Global Vectors for Word Representation.

**Figure 9:**
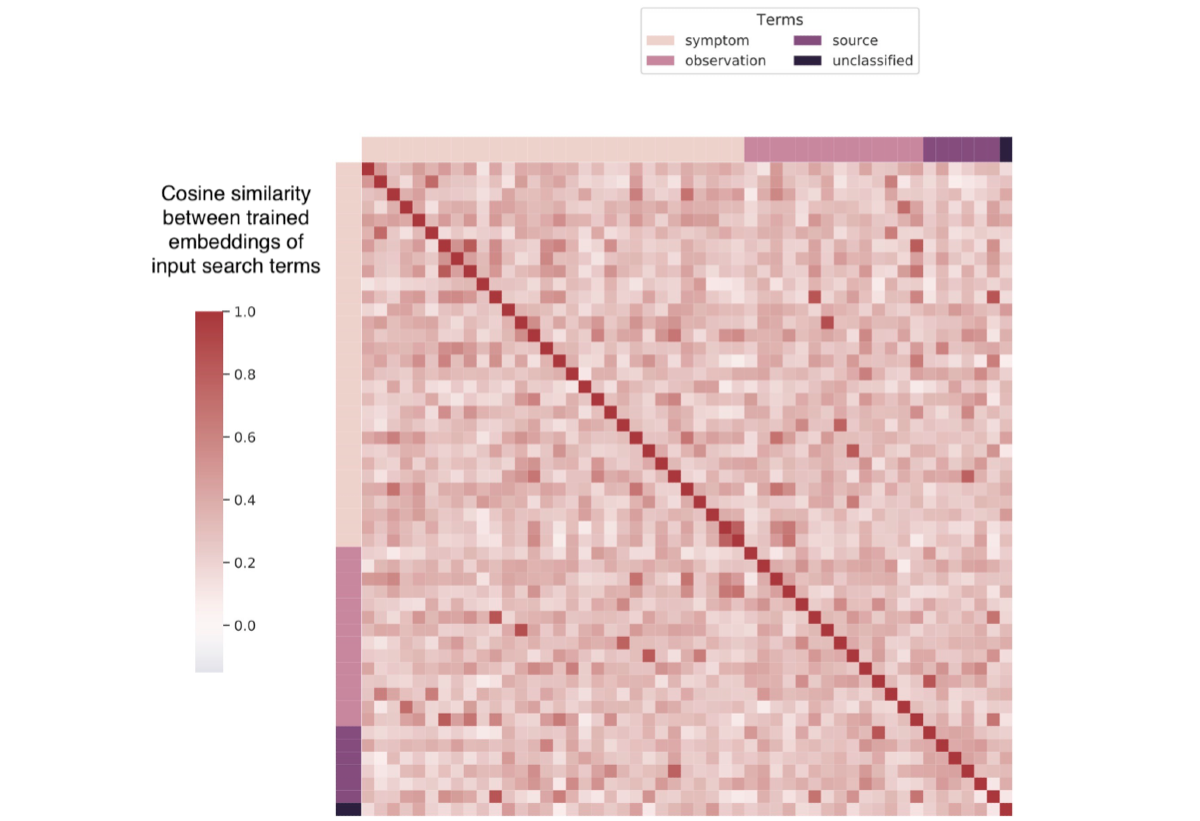
Cosine similarity between trained embeddings of seed search terms.

Compared with existing studies that predict urban air pollution concentrations using linear and nonlinear machine learning models [25,41-47], our proposed method can predict air pollution when source emissions and remotely sensed satellite data are infeasible (eg, sensed satellite data often suffer from a high missing rate owing to frequent cloud cover [48]). Previous studies using web-based search behavior have emphasized the use of Google Trends [40,49] and applied regularized linear regression to collinear web search queries to estimate disease rates from social media or web-based search data [18,19,50-54]. Our research further explored the possibility of using LSTM models with semantic embeddings of search queries to predict air pollution. As shown in Figures 8 and 9, the semantic embeddings of search terms fine-tuned by the DL-LSTM model are less correlated compared with their initial GloVe embeddings, which shows that the collinearity between search terms is reduced during the training process.

We also explored various combinations of search terms and found that a comprehensive set of user queries was critical for accurately capturing people’s responses to urban air pollution. In this study, we expanded the initial set of seed terms using semantic and temporal correlations with search queries from Google Correlate. We investigated the contribution of different search term groups by manually classifying the search terms into 4 categories, where the unclassified category includes terms with ambiguous meanings. Table 8 shows the accuracy and *F*_1_-score when we removed search terms by categories for predicting O_3_, NO_2_, and PM_2.5_ pollution. Removing the search terms in the symptom, observation, and source categories led to a decrease in the accuracy score for detecting at least two pollutants. At the same time, removing the search terms with ambiguous meaning only led to a slightly higher accuracy score for all 3 pollutants.

**Table 8:** Accuracy and *F*1-score of removing different categories of search terms for detecting ozone, nitrogen dioxide, and fine particulate matter pollution using search (dictionary learner-long short-term memory w/search term expansion) as features.

Pollutant and terms			Accuracy (change; %)		*F*_1_-score (change; %)
**O_3_^a^**					
	All	0.6961		0.3244	
	All wo^b^ symptom	0.647 (−7.1)		0.3024 (−6.8)	
	All wo observation	0.622 (−10.6)		0.3264 (+0.6)	
	All wo source	0.6712 (−3.6)		0.3033 (−6.5)	
	All wo unclassified	0.7057 (+1.4)		0.3273 (+0.9)	
**NO_2_^c^**					
	All	0.5684		0.2770	
	All wo symptom	0.4452 (−22.0)		0.2418 (−12.7)	
	All wo observation	0.6125 (+7.8)		0.2480 (−10.5)	
	All wo source	0.5452 (−4.1)		0.2647 (−4.4)	
	All wo unclassified	0.6534 (+15.0)		0.2134 (−23.0)	
**PM_2.5_^d^**					
	All	0.8759		0.0699	
	All wo symptom	0.7897 (−9.8)		0.1029 (+47.2)	
	All wo observation	0.7496 (−14.4)		0.1049 (+50.1)	
	All wo source	0.8994 (+2.7)		0.0393 (−43.8)	
	All wo unclassified	0.8991 (+2.6)		0.0264 (−62.2)	

^a^O_3_: ozone.

^b^wo: without.

^c^NO_2_: nitrogen dioxide.

^d^PM_2.5_: fine particulate matter.

By analyzing the coefficients of each search term, the results show that several search terms contribute more than other search terms. The average feature importance of the seed search terms was calculated using the RF model. As shown in Figure S1, Figure S2, and Figure S3 in Multimedia Appendix 2, search terms including “particular matter,” “rapid breathing,” and “throat irritation” have relatively high feature importance for detecting O_3_, NO_2_, and PM_2.5_ pollution, respectively. The results also indicated that no search terms worked best for all 3 pollutants.

### Limitations

A key limitation of this study is the tuning of the neural network model. First, the performance of neural network models is sensitive to several hyperparameters, including optimization choices, depth, width, and regularization. Owing to computational limitations, we adopted a simple LSTM architecture with a single 128-unit hidden layer and tuned the model using validation data sets for other hyperparameters. In addition, we noticed that stochastic components such as the random seed for the RF model and the randomness in the optimization process of LSTM models influenced the interpretation of the results. Therefore, we repeated the experiments 10 times with different random seeds for the RF and LSTM models. As the time cost of repeating LSTM models is high, we only repeated the RF, LSTM, and DL-LSTM models 10 times to predict O_3_ pollution with all input features. The accuracy of the DL-LSTM model is mean 0.8744 (SD 0.0046). Compared with the LSTM model (mean 0.8714, SD 0.0036), the improvement was not significant (*P*=.11). Compared with the RF model (mean 0.8273, SD 0.0017), the improvement was significant (*P*<.001). The *F*_1_-score for the DL-LSTM model is mean 0.6314 (SD 0.0058). Compared with both the LSTM (mean 0.6019, SD 0.0096) and RF models (mean 0.5588, SD 0.0024), the improvements are significant (*P*<.001), which shows that the results of the LSTM models are stable. There is room for further exploration of more sophisticated neural network model architectures for noninfectious disease prediction [55-57]. We leave the exploration of deeper and wider architectures to future work.

Another limitation relates to the biases introduced by relying on search data, which may not reflect the underlying population demographics or experiences. Although some of these issues are alleviated automatically by training a model against ground sensor pollution levels, understanding and correcting these data biases requires further study. In the future, we plan to investigate other sources of crowd-based surveillance data, such as self-reports on social media, to augment traditional physical sensor methods, thus providing a more direct, human-centered measure of how people experience elevated air pollution levels.

### Conclusions

In this study, we posit that although web search data cannot yet completely replace ground-based pollution monitors, it may already serve as a valuable additional signal to augment ground-based pollution data, providing significant accuracy improvements for detecting unusual spikes in air pollution. We also found that the correlation between search terms and pollution concentration varies at the city level. Therefore, the model must be fine-tuned when applied to specific cities. For model and search term selection, we used the simplest LSTM architecture with a dictionary learner module and found that no search terms worked best for all the 3 pollutants. We propose the use of our model to learn the semantic correlations between available search terms to obtain better prediction results.

## Appendix

Multimedia Appendix 1 The descriptions of search terms, data source, and model hyperparameters.

Multimedia Appendix 2 Average feature importance for detecting ozone, nitrogen dioxide, and fine particulate matter pollution using random forest models.

## Abbreviations

DL-LSTM: dictionary learner-long short-term memory
GloVe: Global Vectors for Word Representation
LR: logistic regression
LSTM: long short-term memory
MSA: metropolitan statistical area
NO_2_: nitrogen dioxide
O_3_: ozone
PM_2.5_: fine particulate matter
RF: random forest
RNN: recurrent neural network
STE: search term expansion
