# Supplementary material for: Detecting Elevated Air Pollution Levels by Monitoring Web Search Queries: Algorithm Development and Validation
Source: JMIR Form Res. 2022 Dec 19;6(12):e23422. doi: 10.2196/23422 (PMC9808603; doi:10.2196/23422)
Supplement: Multimedia Appendix 1 [file formative_v6i12e23422_app1.pdf]

Appendix 1

Table S1. Categories of seed search terms

Category	Terms
Symptom	chest tightness, respiratory infection, copd, throat irritation, rapid heart rate, wheezing, cough,snoring, chest pain, pediatric asthma, rapid pulse, respiratory illness, coughing, cardiovascular disease, shortness of breath, childhood asthma, arrhythmia, rapid breathing, headache, inhaler use, asthma attack, asthma, difficulty breathing, bronchitis, heart murmur, chronic obstructive pulmonary disease, inhaler, lung cancer, irregular heartbeat, lung disease
Observation	black carbon, smoggy, nitrogen dioxide, code orange, code red, air pollution, particulate matter, organic carbon, haze,smog, air pollutant, ozone, smoke, sulfate
Source	industrial pollution, tailpipe, soot, traffic, wildfires, power plants
Unclassified	premature death

Table S2. Categories of expanded search terms

Category	Terms
Symptom	advair, albuterol, allergies, allergy, apnea, asthma attacks, asthmatic, bad health, being asleep, breathing, chest pains, chronic lung disease, coughing wheezing, coughs, croup, diarrhea,dizziness, dry cough, cough runny nose, coughed, eczema, emphysema, fog, foggy, gasping, hacking cough, heart arrhythmia, hypertension, insomnia, interstitial pneumonia, labored breathing, metered dose inhaler, nausea,persistent cough, phlegm, respiratory, respiratory disease, respiratory distress syndrome, runny, runny nose, salbutamol, shortness, sleep,sneezing, sneezing watery eyes, sniffling, snore, snored, snorer, snores,symptoms, throat, tinnitus, ventolin, vomiting, wheeze, wheezes, wheezy
Observation	ash, carbon black, clouds, code black, code blue, code brown, code pink, dust, gases, hazes, hazy, inhalation, inhalers, inhaling, mist, mists, misty, monoxide, ozone layer, ozone pollution, ozone smog, particulate, pollutant, pollutants, polluted air, pollution, smokes, sooty, vapors
Source	emissions, flames, from fire, fumes, gun smoke, nebulizer, obstructive, sulfur, thermal pollution
Unclassified	find match, light cigarette, secondhand smoke, second hand smoke, smoking

SEARCH TERMS

The search terms consist of 51 seed terms suggested by domain experts and 101 expanded terms by exploring the semantic and temporal correlations between seed terms and the user search queries from Google Correlate. The seed terms and expanded terms are listed in detail in Table S1 and Table S2.

DATA SOURCE

We utilize two sources of data, namely search query volumes from Google Trends and pollutant levels from the United States Environmental Protection Agency (EPA).

Search Query Volumes

Time series of daily search frequency were retrieved from Google Trends. Search frequency represents the search activity of a query within a geographical region. The frequency of a query "is divided by the total searches of the geography and time range it represents to compare relative popularity. The resulting numbers are then scaled on a range of 0 to 100 based on a topic's proportion to all searches on all topics.

We approach the task as a multivariate time series problem. When multiple keywords are queried as a set, the keyword with the highest frequency downscales the other, less frequent, keywords. Since our model leverages daily deviations in search frequency, we query each keyword individually to preserve the most information.

EPA Data

We obtained pollution data for Ozone, Nitrogen Dioxide and Particulate Matter 2.5 and meteorological data for temperature and relative humidity from EPA website. Each daily observation uses the median observed value across multiple sensor sites with the units of measurement as follows, ozone takes the highest observed 8-hour average measured in parts per million (ppm), nitrogen dioxide takes the highest observed 1-hour average measured in parts per billion and PM2.5 takes daily average measured in micrograms/cubic meter, temperature in Farenheit, and relative humidity as a percentage. Our environmental data spans the same time range as our search query frequency, January 1, 2007 to December 31, 2018 inclusive.

Model Hyper-parameter

Table S4. Hyper-parameters for LR + Elastic Net, RF and LSTM & DL-LSTM models

**Model**	**Hyper-parameter**
LR + Elastic Net	Cs: [e^-4, e^-3, e^-2, e^-1, 1, e, e^2, e^3, e^4]<br>l_1_ratios: [e^-8, e^-7, e^-6, e^-5,e^-4, e^-3, e^-2, e^-1, 1]
RF	n_estimators: [50, 100, 150, 300, 450]<br>max_depth: [3,5,7,9]
LSTM & DL-LSTM	batch_size: 32<br>learning_rate: 0.0001<br>optimizer: Adam
