# Supplementary material for: Detecting Elevated Air Pollution Levels by Monitoring Web Search Queries: Algorithm Development and Validation
Source: JMIR Form Res. 2022 Dec 19;6(12):e23422. doi: 10.2196/23422 (PMC9808603; doi:10.2196/23422)

Appendix 2.

Figure S1. Average feature importance for detecting ozone pollution using Met+Search (random forest) model. Met: meteorological data.

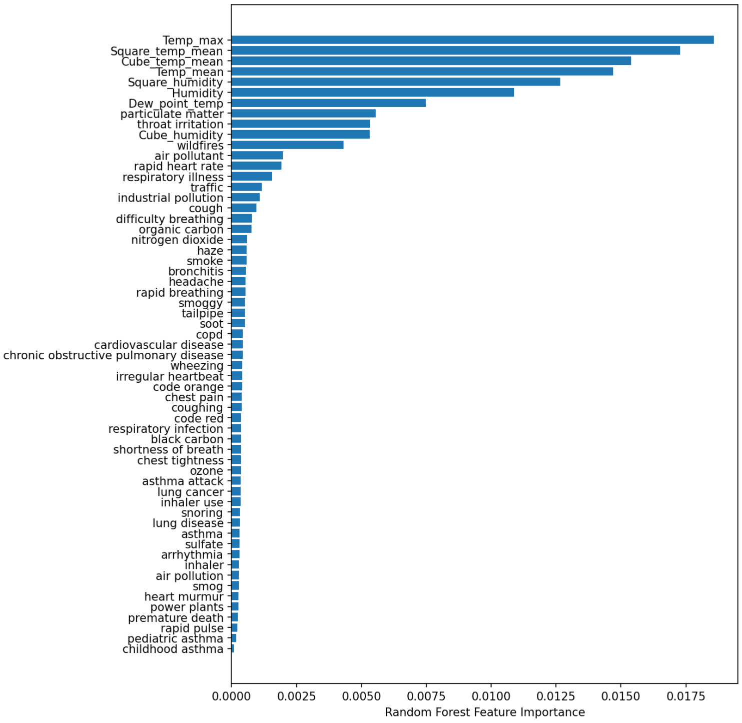

Figure S2. Average feature importance for detecting nitrogen dioxide pollution using Met+Search (random forest) model. Met: meteorological data.

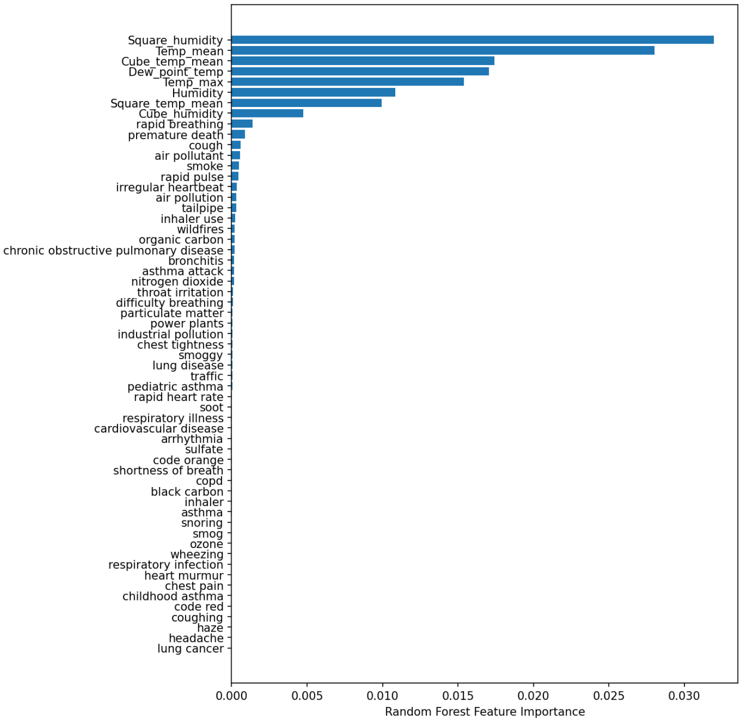

Figure S3. Average feature importance for detecting fine particulate matter pollution using Met+Search (random forest) model. Met: meteorological data.

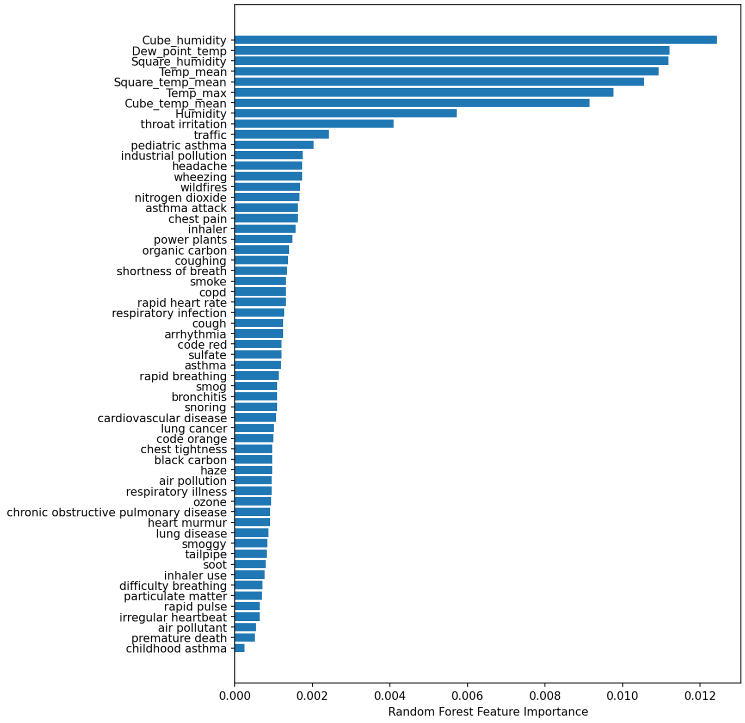

Supplement: Multimedia Appendix 2 [file formative_v6i12e23422_app2.pdf]
